# Analysis of the Anti-Inflammatory and Anti-Osteoarthritic Potential of Flonat Fast^®^, a Combination of *Harpagophytum Procumbens* DC. ex Meisn., *Boswellia Serrata* Roxb., *Curcuma longa* L., Bromelain and Escin (*Aesculus hippocastanum*), Evaluated in In Vitro Models of Inflammation Relevant to Osteoarthritis

**DOI:** 10.3390/ph15101263

**Published:** 2022-10-13

**Authors:** Stefano Quarta, Giuseppe Santarpino, Maria Annunziata Carluccio, Nadia Calabriso, Egeria Scoditti, Luisa Siculella, Fabrizio Damiano, Michele Maffia, Tiziano Verri, Raffaele De Caterina, Marika Massaro

**Affiliations:** 1 Department of Biological and Environmental Sciences and Technologies (DISTEBA), University of Salento, 73100 Lecce, Italy; 2 Cardiovascular Center, Paracelsus Medical University, 90471 Nuremberg, Germany; 3 GVM Care & Research, Città di Lecce Hospital, 73100 Lecce, Italy; 4 Cardiac Surgery Unit, Department of Experimental and Clinical Medicine, University “Magna Graecia”, 88100 Catanzaro, Italy; 5 Institute of Clinical Physiology (IFC), National Research Council (CNR), 73100 Lecce, Italy; 6 Cardiology Division, Pisa University Hospital, 56126 Pisa, Italy; 7 Fondazione VillaSerena per la Ricerca, Città Sant’Angelo, 65013 Pescara, Italy

**Keywords:** inflammation, angiogenesis, monocyte recruitment, osteoarthritis, plant bioactives, antioxidants, nutritional supplements, immune response

## Abstract

Osteoarthritis (OA) is a joint disease characterized by inflammation of the synovium, angiogenesis, cartilage degradation, and osteophyte formation. *Harpagophytum Procumbens* DC. ex Meisn., *Boswellia Serrata* Roxb., *Curcuma longa* L., Bromelain and Escin (*Aesculus hippocastanum*) are plants which extracts, together to Bromelain and Escin (*Aesculus hippocastanum*) are traditionally used in OA. However, their mechanistic role remains unclear. We aimed to investigate whether these bioactives alone or in combination (as in Flonat Fast^®^) can suppress TNF-α-induced inflammation, angiogenesis, and osteophyte formation using two cell models involved in OA: endothelial cells and monocytes. Each plant extract was evaluated for its polyphenol content, antioxidant activity, and toxicity. In endothelial cells and monocytes, expression of genes involved in OA was assessed, functional assays for inflammation and angiogenesis were performed, and impairment of reactive oxygen species production (ROS) was evaluated. Exposure of cells to the bioactives alone and in combination before cytokine stimulation resulted in differential counterregulation of several gene and protein expressions, including those for cyclooxygenases-2, metalloproteinase-9, transforming growth factor β1, and bone morphogenic protein-2. We demonstrated that these bioactives modulated monocyte adhesion to endothelial cells as well as cell migration and endothelial angiogenesis. Consistent with radical scavenging activity in the cell-free system, the bioactives curbed TNF-α-stimulated intracellular ROS production. We confirmed the potential anti-inflammatory and antiangiogenic effects of the combination of *Harpagophytum procumbens*, *Boswellia*, Curcuma, Bromelain, and Escin and provided new mechanistic evidence for their use in OA. However, further clinical studies are needed to evaluate the true clinical utility of these bioactives as supportive, preventive, and therapeutic agents.

## 1. Introduction

Osteoarthritis (OA) is a degenerative pro-inflammatory joint disease characterized by cartilage degradation and synovial inflammation, accompanied by pro-inflammatory angiogenesis and osteophyte formation [1]. Synovial inflammation appears to play a critical role in the pathogenesis of OA, as evidenced by increased numbers of bone lining cells and by the inflammatory infiltrate consisting primarily of monocytes and their downstream progeny, such as osteoclasts and synovial macrophages [2]. Although the temporal sequence of OA initiation remains unclear, prolonged mechanical stress is thought to trigger synovitis by damaging subchondral bone and articular cartilage that release extracellular matrix fragments [3]. These degradation products are indeed known to promote inflammation and trigger the release of various pro-inflammatory cytokines in resident cells [4]. This leads to an exacerbation of synovitis through the attraction, recruitment, and influx of circulating monocytes [4]. In the appropriate microenvironment, the migrated monocytes differentiate into synovial macrophages [4] and/or osteoclasts [5] and cause inflammatory edema and joint effusion [6] or bone resorption and joint destruction [7]. Synovial macrophages activated by pro-inflammatory cytokines and products of cartilage degradation [4,8] respond by releasing vascular endothelial growth factor (VEGF), tumour necrosis factor (TNF)-α, Interleukin(IL)-1β, IL-6, and various chemokines such as monocyte chemoattractant protein (MCP)-1, which can stimulate sprouting of new leaky capillaries and further promote recruitment of other circulating leukocytes [4,8,9]. In this pro-inflammatory milieu, vascular endothelial cells are induced to switch to a pro-adhesive and pro-inflammatory phenotype and in this way increase the expression of pro-inflammatory cytokines and enzymes such as cyclooxygenase (COX)-2 (target of non-steroidal anti-inflammatory drugs (NSAIDs)), thereby fueling all pro-inflammatory symptoms of OA, including pain and swelling [1]. The overproduction of cytokines and growth factors from the inflamed synovium also induces the release of matrix metalloproteinases (MMPs) [9]. These proteolytic enzymes are involved in tissue destruction in both inflammatory and degenerative joint disease [10]. Elevated levels of MMP-2 and MMP-9 have been detected in the synovial fluid of osteoarthritic human cartilage [11,12]. Furthermore, in some forms of OA, MMP-9 levels in plasma and serum have been shown to correlate positively with the severity of damage to articular cartilage and subchondral bone [13]. Although current pharmacotherapy offers several options for the relief of pain and other symptoms of OA (including NSAIDs, acetaminophen, tramadol), concerns about safety in chronic treatments and cost have sparked interest in natural remedies. Flonat Fast^®^ is a nutraceutical product that combines the anti-inflammatory and anti-arthritic properties of five different traditional plant extracts: bromelain (B) [14]; *Harpagophytum procumbens* DC. ex Meisn., (HP), also known as Devil’s Claw [15]; *Boswellia serrata* Roxb (BS) [14]; *Curcuma longa* L. (C), rich in curcuminoids, especially curcumin [16]; and cortex of *Aesculus hippocastanum* L., rich in escin (E) [17]. Given the limited pharmacological treatment options to combat this complex multifactorial and multicellular pathology, the discovery of dietary supplements that can be used to provide long-term symptom relief in people with OA may represent an important therapeutic achievement. We hypothesize that the combination of B, HP, BS, C, and E could enhance their respective beneficial actions and more efficiently influence the inflammatory stigmata of OA. With this in mind, the objective of this study was to evaluate the ability of the Flonat Fast^®^ components, alone and in combination, to inhibit several critical pro-inflammatory features of OA pathogenesis, including monocyte and endothelial inflammation, monocytes recruitment, pro-inflammatory angiogenesis, and osteophyte formation, as potential mechanistic evidence of the utility of this bioactive combination as a supportive tool in current therapeutic approaches to OA.

## 2. Results

### 2.1. Total Polyphenol Content and Radical Scavenging Effects

All bioactives that make up Flonat Fast^®^ were independently analysed for their total phenolic content (TPC). After solubilization of B, HP, and E in water, BS in DMSO and C in ethanol, the TPC of each solution was determined by Folin–Ciocalteau assay. The results are summarised in Table 1. As expected, the highest concentrations of polyphenols were found in the extracts of C (694.51 ± 132.58 µg GAE/mg dry extract) and E (136.6 µg GAE/mg dry extract). Lower values were found for BS (43.79 ± 6.81 µg GAE/mg dry extract). The lowest values were found for B and HP (about 7 µg GAE/mg dry extract). Since polyphenolic compounds contribute mechanistically to the antioxidant capacities of plant bioactives, we further investigated the antioxidant activity of each bioactive by the ABTS assay. We did not find close agreement with the TPC, as the greatest antioxidant activity was found for E, followed by C and then BS with 11.6 and 7.7 µmol TE/mg dry extract, respectively (Table 1).

### 2.2. Effect of B, HP, BS, C, and E Alone and in Combination on Monocytes and Endothelial Cell Viability

**Figure 1:**
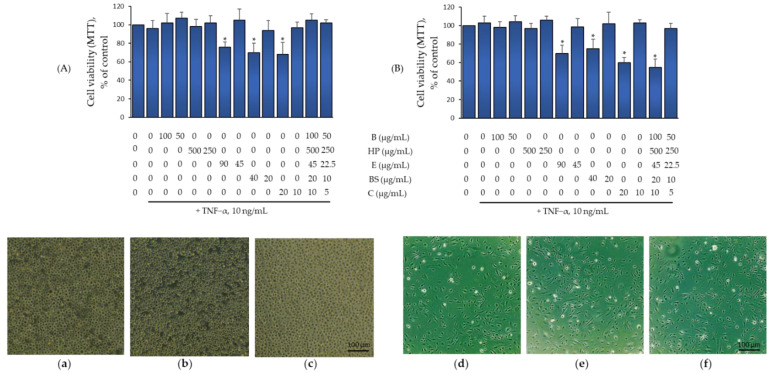
The effect of Flonat Fast^®^ (FF) components treatment on cell viability. (**A**) THP-1 were treated with FF components for 4 h at the concentrations indicated, and then either treated with 10 ng/mL TNF-α or left untreated for 18 h. Cell viability was assessed by the 3-(4,5dimethylthiazolyl)-2,5-dipgenyl-tetrazolium bromide (MTT) assay and expressed as percent of basal (untreated) control. Data (means ± S.D., *n* = 3) are expressed as percent of unstimulated control. * *p* < 0.01 vs. basal (untreated) control. Images were visualized and acquired with a phase contrast microscope at 10× magnification. (**a**) control; (**b**) TNF-α 10 ng/mL; (**c**) FF + TNF-α. (**B**) HMEC-1 were treated with FF components for 4 h at the concentrations indicated, and then either treated with 10 ng/mL TNF-α or left untreated. Cell viability was assessed by the 3-(4,5dimethylthiazolyl)-2,5-dipgenyl-tetrazolium bromide (MTT) assay and expressed as percent of unstimulated control. Data (means ± S.D., *n* = 3) are expressed as percent of basal (untreated) control. * *p* < 0.01 vs. basal (untreated) control. Images were visualized and acquired with a phase contrast microscope at 10× magnification. (**d**) control; (**e**) TNF-α 10 ng/mL; (**f**) FF + TNF-α.

Each compound under investigation was tested for cell toxicity at concentrations corresponding to those potentially achievable in vivo (10–500 µg/mL: E [18], B [19], C [20], HP [21], and BS [22].), individually and in combination both in the presence or in the absence of TNF-α. After cell treatment, viability was monitored by examining the residual ability of cells to convert MTT to formazan crystals, the total protein content of cells, and the change in cell shape by phase contrast microscopy. As shown in Figure 1A (upper panel), treatment of THP-1 with E, BS, and C at higher concentrations proved toxic. For this reason, when the compounds were combined, we evaluated the higher non-toxic concentrations as such and at a half of the concentrations of the individual components alone. Under both conditions, the combination of B (100 and 50 µg/mL), HP (500 and 250 µg/mL), BS (20 and 10 µg/mL), C (10 and 5 µg/mL), and E (45 and 22.5 µg/mL) had no effect on cell viability, either in the absence or presence of TNF-α (Figure 1A). At the same time, morphological examination under phase-contrast microscopy for mixed compounds at higher concentrations showed no change in cell morphology (Figure 1A, lower panels). In close analogy to THP-1, treatment of HMEC-1 with E, BS, and C also proved toxic at the highest concentrations (Figure 1B, upper panel). For this reason, we tested in combination the higher, non-toxic concentrations as such and halved. In contrast to THP-1, HMEC-1 was more sensitive to bioactives at higher concentrations. However, when each bioactive was added at halved concentrations (B, 50 µg/mL; HP, 250 µg/mL; BS, 10 µg/mL; C, 5 µg/mL; and E, 22.5 µg/mL), the mixture of bioactives had no effect on cell viability, both in the absence and presence of TNF-α. At the same time, morphological examination under phase contrast microscopy showed no change in cell morphology or loss of adherence potential (Figure 1B, lower panels). Therefore, these were the highest concentrations tested in the following anti-inflammatory assays and cell migration studies. 

### 2.3. Comparison of the Activity of B, HP, BS, C, and E on Gene and Protein Expression of IL-6, IL-8, MMP-9, and COX-2 in TNF-α-Stimulated Human Monocytes

**Figure 2:**
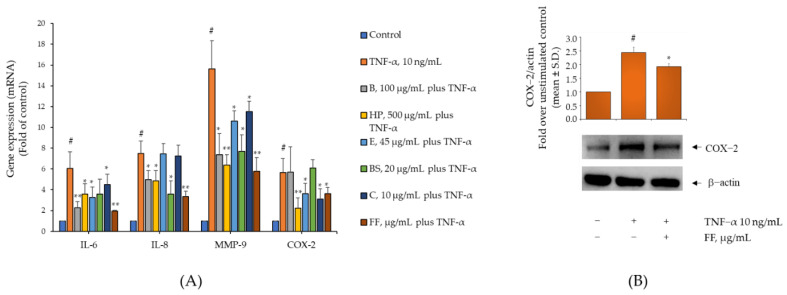
Flonat Fast^®^ components attenuate TNF-α-induced monocytes inflammation. THP-1 were treated with FF components (B, 50 µg/mL; HP, 250 µg/mL; E, 22.5 µg/mL; BS, 10 µg/mL; and C, 5 µg/mL) for 4 h and then either treated with 10 ng/mL TNF-α or left untreated for 5 h. (**A**) Total RNA was extracted from cells, and mRNA levels of IL-6, IL-8, MMP-9, and COX-2 were measured by qPCR using specific primers and normalized to 18S RNA. Data (means ± S.D., *n* = 3) are expressed as fold induction over basal (untreated) control. # *p* < 0.01 vs. basal (untreated) control; * *p* < 0.05 vs. TNF-α alone; ** *p* < 0.01 vs. TNF-α alone. (**B**) Whole-cell lysates were assayed by Western blotting using antibodies against COX-2 and β-actin. Intensity of protein bands were quantified by densitometry and results (means ± SD, *n* = 3), expressed as COX-2/β-actin, were presented as percentage of basal (untreated) control. # *p* < 0.01 vs. basal (untreated) control; * *p* < 0.05 vs. TNF-α alone.

TNF-α has been shown to play a central role in orchestrating the inflammatory processes underlying OA development [23]. To investigate whether Flona Fast^®^ components B, HP, BS, C, and E were able to exert anti-inflammatory effects and attenuate pro-inflammatory stigmas mediated by monocyte/macrophage activities in the inflamed synovium [24], THP-1 were exposed to each Flonat Fast^®^ component alone or in combination for 4 h and then stimulated with TNF-α for an additional 5 h. Thereafter, gene expressions of the pro-inflammatory chemokines IL-6 and IL-8, the matrix-degrading enzyme MMP-9, and the pro-inflammatory enzyme COX-2 were determined by real-time PCR. As shown in Figure 2A, cell exposure to TNF-α alone significantly induced the expression of all tested genes (*p* > 0.001). Induction of IL-6 was counterregulated by B, HP, C, and E, but not by BS. Induction of IL-8 was counterregulated by B, HP, and BS, but not by C and E. Both chemokines were downregulated to a greater extent when the Flonat Fast^®^ components were administered in combination at half of the concentrations of the individual components alone. Induction of MMP-9 was downregulated by each of the Flonat Fast^®^ components alone and again to a greater extent by their combination at halved concentrations. Similarly, induction of COX-2 was counterregulated by HP, C, and E, but not by B and BS. Moreover, when cells were exposed to the combination of all Flonat Fast^®^ components at halved concentration, expression of COX-2 was also significantly reduced, affecting gene expression and protein expression (Figure 2B). Overall, these data support the potential comprehensive (multi-genic) anti-inflammatory effect of the Flonat Fast^®^ components when administered in combination.

### 2.4. Comparison of B, HP, BS, C, and E Activity in Relation to Endothelial Expression of Genes and Proteins Involved in Inflammation, Leukocyte Adhesion, and Migration

**Figure 3:**
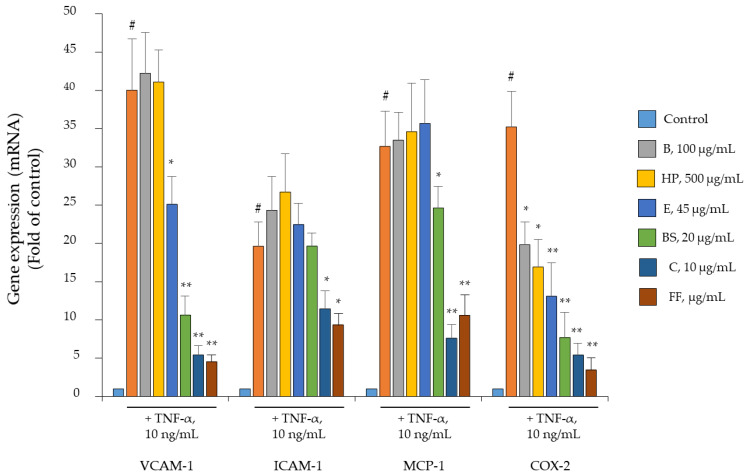
Flonat Fast^®^ components counteract TNF-α-induced expression of inflammatory gene in human endothelial cells. HMEC-1 were treated with FF components alone at the indicated concentration or in combination (at halved concentration: B, 50 µg/mL; HP, 250 µg/mL; E, 22.5 µg/mL; BS, 10 µg/mL; and C, 5 µg/mL) for 4 h and then either treated with 10 ng/mL TNF-α or left untreated for 18 h. Total RNA was extracted from cells, and mRNA levels of VCAM-1, ICAM-1, MCP-1, and COX-2 were measured by qPCR using specific primers and normalized to GAPDH RNA. Data (means ± S.D., *n* = 3) are expressed as fold induction over basal (untreated) control. # *p* < 0.01 vs. basal (untreated) control; * *p* < 0.05 vs. TNF-α alone; ** *p* < 0.01 vs. TNF-α alone.

The importance of inflammation and angiogenesis in both OA and rheumatoid arthritis (RA) has been underscored by evidence that the synovium of OA and RA patients has a pink or even red appearance due to the increased number of inflamed blood vessels [25]. Therefore, we investigated in detail the ability of Flonat Fast^®^ components to inhibit endothelial inflammation and its pro-adhesive and pro-angiogenic consequences. As shown in Figure 3, the expression of the typical pro-adhesive genes VCAM-1 and ICAM-1 as well as the chemokine MCP-1 was strongly induced under TNF-α stimulation (*p* < 0.001). However, when cells were exposed to each single Flonat Fast^®^ component, only E, BS, and C were able to downregulate the induction of tested genes. Contrariwise, when the components were administered together, the expression of each gene was counterregulated to a greater extent than with each component alone, although they were combined at halved concentrations. Interestingly, the induction of COX-2 was counterregulated by each component and to a greater extent when the components were administered together, which is consistent with the other pro-inflammatory genes tested. 

**Figure 4:**
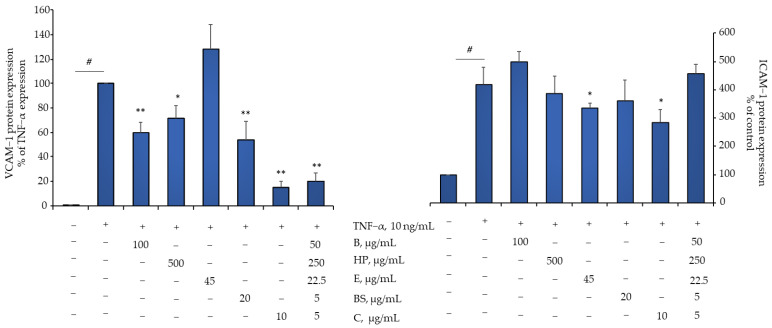
Flonat Fast^®^ components reduce TNF-α-induced expression of endothelial cells adhesion molecules. HMEC-1 were treated with FF components (4 h) and then either treated with 10 ng/mL TNF-α for 18 h or left untreated. Endothelial cell surface expression of VCAM-1 and ICAM-1 was assessed by cell surface EIA and expressed as percentage of unstimulated control. Data (means ± S.D., *n* = 3) are expressed as fold induction over basal (untreated) control for ICAM-1 and as fold induction over TNF-α for VCAM-1. # *p* < 0.01 vs. basal (untreated) control; * *p* < 0.05 vs. TNF-α alone; ** *p* < 0.01 vs. TNF-α alone.

Remarkably, we observed that the activities of the Flonat Fast^®^ components in modulating gene transcriptional regulation were mirrored by significant counterregulation of protein expression for some of the genes tested, including the expression of VCAM-1 and, to a lesser extent, ICAM-1, two key players in controlling the pro-adhesive properties of the endothelium in OA (Figure 4). 

**Figure 5:**
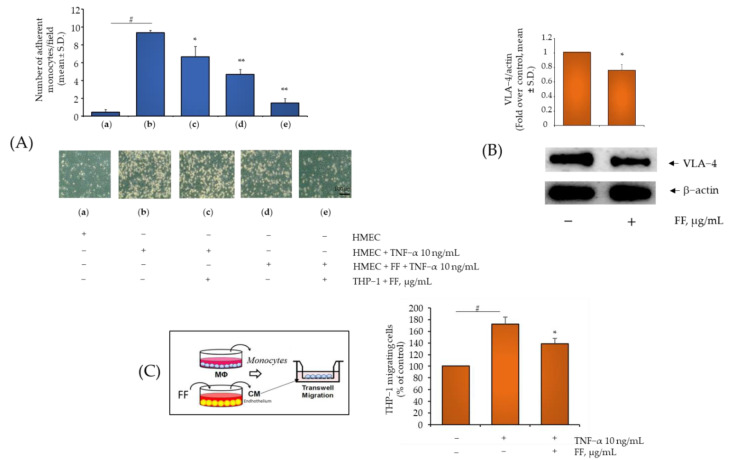
Flonat Fast^®^ components attenuate TNF-α-induced endothelial monocytes adhesion and chemiotaxis. (**A**) HMEC-1 were treated with Flonat Fast^®^ components (B, 50 µg/mL; HP, 250 µg/mL; E, 22.5 µg/mL; BS, 10 µg/mL; C, 5 µg/mL) (4 h) and then either treated with 10 ng/mL TNF-α or left untreated for 18 h. THP-1 were treated with Flonat Fast^®^ components (4 h) or left untreated. Treated and untreated THP-1 (10^6^ cells/mL) were added to the HMEC-1 monolayers. After 1 h, non-adhering cells were removed by three washes and images of HMEC-1 and adherent THP-1 cells were visualized and captured with a phase contrast microscope at 10× magnification. Data (means ± S.D., *n* = 3) are expressed as number of adherent monocytes per fields. # *p* < 0.01 vs. basal (untreated) control; * *p* < 0.05 vs. TNF-α alone; ** *p* < 0.01 vs. TNF-α alone. (**B**) THP-1 were treated with FF component (4 h) or left untreated. Whole-cell lysates were assayed by Western blotting using antibodies against VLA-4 and β-actin. Intensity of protein bands were quantified by densitometry and results (means ± SD, *n* = 3), expressed as VLA-4/β-actin, were presented as percentage of basal (untreated) control. * *p* < 0.05 vs. basal (untreated) control. (**C**) HMEC-1 were treated with Flonat Fast^®^ components (4 h) and then either treated with 10 ng/mL TNF-α for 18 h or left untreated. Culture medium were collected and added to the lower chamber in a Boyden chamber. THP-1 (2.5 × 106 cells/mL) were added to the upper chamber. After 30 min, migrated THP-1 cells were measured by MTT assay. Data (means ± S.D., *n* = 3) are expressed as number of migrated monocytes. # *p* < 0.01 vs. basal (untreated) control; * *p* < 0.05 vs. TNF-α alone.

Since preventing monocyte attraction and adhesion in the inflamed synovium could be an additional therapeutic target to slow down the exacerbation of OA, we investigated the potential functional effects of downregulation of endothelial adhesion molecule expression and chemotactic factors by Flonat Fast^®^ components by performing both adhesion assays between endothelial cells and monocytes and transwell cell migration assays. As a consequence of the global and profound reprogramming of pro-inflammatory gene expressions, TNF-α massively supported the adhesion of monocytes to the endothelial surface (Figure 5A, panel 2 versus 1 and Supplemental Figure S1). 

Consistent with the anti-inflammatory effect of Flonat Fast^®^ expressed as a reduction of stimulated endothelial expression of adhesion molecules and chemoattractants, cell exposure to HP, BS, C, and E, but not B, significantly reduced monocyte adhesion (Supplemental Figure S1). As expected, exposure of endothelial cells to combined Flonat Fast^®^ components resulted in greater inhibition of monocyte adhesion (Supplemental Figure S1, panel 8 versus panel 2). The same functional return was observed when monocytes were exposed to Flonat Fast^®^ components. As shown in Figure 5A, exposure of only monocytes to Flonat Fast^®^ was able to reduce monocyte adhesion to inflamed endothelium by approximately 30% (panel 3 vs. panel 2, *p* < 0.05). However, when both endothelial cells and monocytes were treated with Flonat Fast^®^ components, the inhibition of adhesion rates reached 80% (panel 5 vs. panel 2, *p* < 0.001). To address such effects we also evaluated the effect of Flonat Fast^®^ components on the expression of the integrin VLA-4, constitutively expressed on the cell surfaces of T and B cells and monocytes and functionating as a promotor of inflammatory response by assisting the movement of leukocytes to inflamed tissue [26], and observed a significant reduction of VLA-4 protein expression (Figure 5B). These data indicate the potential broad-spectrum activity of Flonat Fast^®^ components when combined. Next, we demonstrated the potential ability of FonatFast^®^ to affect monocyte chemoattraction to inflamed endothelium. To this end, we treated endothelial cell monolayers with the combination of the Flonat Fast^®^ components before stimulation with TNF-α. After 24 h, the endothelial medium was collected and added to the lower chamber of a transwell system, while the monocytes were added to the upper chamber. After 4 h, monocytes that had migrated to the lower chamber were measured. Consistent with a decreased release of chemoattractant molecules by endothelial cells exposed to Flonat Fast^®^ components, we observed a significant reduction in the number of migrated monocytes (Figure 5C). 

### 2.5. Comparison of B, HP, BS, C, and E Activity on the Endothelial Expression of Gene and Protein Involved in Angiogenesis and Osteophyte Formation

**Figure 6:**
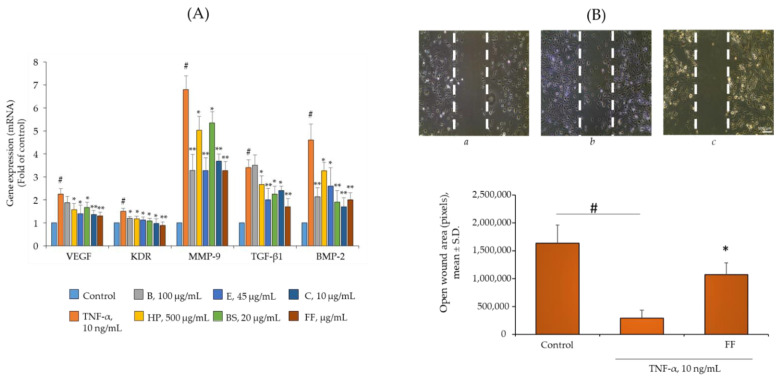
Flonat Fast^®^ components reduce TNF-α-induced angiogenesis and expression of pro-osteogenesis genes in human endothelial cells. (**A**) HMEC-1 were treated with FF components alone at the indicated concentration or in combination (at halved concentration: B, 50 µg/mL; HP, 250 µg/mL; E, 22.5 µg/mL; BS, 10 µg/mL; and C, 5 µg/mL) for 4 h and then either treated with 10 ng/mL TNF-α or left untreated for 18 h. Total RNA was extracted from cells, and mRNA levels of VEGF, KDR, MMP-9, TGB-β1, and BMP-2 were measured by qPCR using specific primers and normalized to GAPDH RNA. Data (means ± S.D., *n* = 3) are expressed as fold induction over basal (untreated) control. # *p* < 0.01 vs. basal (untreated) control; * *p* < 0.05 vs. TNF-α alone; ** *p* < 0.01 vs. TNF-α alone. (**B**) A scratch wound was performed on endothelial monolayers of HMEC-1 and then cells were treated with FF components for 4 h and then either treated with 10 ng/mL TNF-α or left untreated for 18 h. Cell migration was quantified and monitored under phase-contrast microscopy (10× magnification) after 18 h. Data (means ± S.D., *n* = 3) are expressed as open wound area. # *p* < 0.01 vs. basal (untreated) control; * *p* < 0.05 vs. TNF-α alone.

Because disruption of the vascular network due to sprouting of new fragile and leaky capillaries may facilitate monocyte extravasation [27], we also investigated the antiangiogenic effect of Flonat Fast^®^ components by measuring the expression of key proangiogenic genes, such as VEGF, KDR, and MMP-9, in addition to COX-2. As shown in Figure 6A, TNF-α was also able to induce the expression of VEGF, KDR, and MMP-9. In close analogy to the modulation of the other pro-inflammatory genes tested, Flonat Fast^®^ components alone and in combination at halved concentrations decreased the expression of MMP-9, KDR, and VEGF. Only B showed no effect in downregulating VEGF gene expression. 

During angiogenesis, cell motility and migration are critical steps to ensure vessel growth. The potential anti-angiogenic activity of Flonat Fast^®^ suggested by the downregulation of pro-angiogenic gene expression was confirmed by a functional angiogenesis assay, the scratch assay, which measures the acquisition of motility properties by endothelial cells when contact inhibition is lost and cells are stimulated by pro-inflammatory agents such as TNF-α [28]. As shown in Figure 6B, endothelial exposure to TNF-α in conjunction with cell detachment significantly increased the migration rate of HMEC-1 toward the acellular wound area compared with the migration rates of the same cells under control conditions (Figure 6, panel *b* versus panel *a*), resulting in an 80% increase in the area covered by cells (*p* < 0.01). Pretreatment of cells with Flonat Fast^®^ components slowed TNF-α-stimulated endothelial cell migration, with a mean increase in cell-covered area of only 34% (*p* < 0.05).

Angiogenesis may also promote chondrocyte hypertrophy and endochondral ossification, contributing to the radiographic changes in the joint [29]. We therefore examined endothelial gene expression of TGF-β and BMP-2. As shown in Figure 6A, TNF-α induced expression of both genes, but pretreatment of endothelial cells with Flonat Fast^®^ components reversed the effect of TNF-α and downregulated the induction of TGF-β and BMP-2.

### 2.6. Effect of the Combination of Flonat Fast^®^ Components on Monocytes and Endothelial Dysregulation in ROS Production

Given the chemical nature of the compounds tested, which have shown inherent antioxidant activity (see Table 1), the most plausible mechanism of action could be the curbing of the excessive production of ROS triggered by cell stimulation with TNF-α.

**Figure 7:**
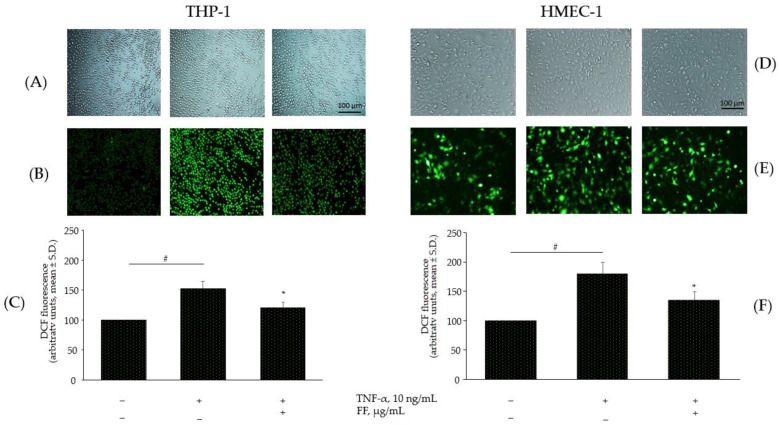
Flonat Fast® components counteract TNF-α-induced ROS production. Monocytes (THP-1), and endothelial cells, (HMEC-1) were treated with FF in combination ((**B**), 50 µg/mL; HP, 250 µg/mL; (**E**), 22.5 µg/mL; BS, 10 µg/mL; and (**C**), 5 µg/mL) for 4 h and then either treated with 10 ng/mL TNF-α or left untreated for 20 min. Intracellular ROS were analysed by using car-boxy-H2DCFDA staining. (**A**,**B**,**D**,**E**). Images were visualized and captured with a fluorescent stereomicroscope. (**C**,**F**) Fluorescence was also measured by a fluorescence plate reader. Data (means ± S.D., *n* = 3) are expressed as percentage of intracellular ROS levels of basal (untreated) control. # *p* < 0.01 vs. basal (untreated) control; * *p* < 0.05 vs. TNF-α alone; 10× magnification.

We therefore tested the effect of Flonat Fast^®^ components on TNF-α-induced intracellular ROS accumulation in monocytes and endothelial cells. In both cell models, basal intracellular production of ROS, as measured by dichlorofluorescein fluorescence, was significantly increased by exposure to TNF-α. Preincubation with Flonat Fast^®^ components at the indicated concentrations for 4 h before cytokine stimulation decreased the induced production of ROS. Qualitative examples are shown in Figure 7A,B, and quantitative data are shown in Figure 7C. Flonat Fast^®^ components decreased TNF-α-induced intracellular production of ROS by 25% in THP-1 (*p* < 0.05) and by 21% in HMEC-1 (*p* < 0.05).

## 3. Discussion

We have characterized here the potential anti-osteoarthritic effect of a combination of antioxidant plant bioactives that have shown the ability to prevent and/or reduce inflammation and associated stigmas in two different but closely interacting cell models, monocytes and endothelial cells. 

Although OA has long been classified as a non-inflammatory joint disease and pathogenetically characterized by mechanical damage and cartilage loss, it has recently been viewed as a multifactorial disease for which there is no single cause and no precise etiology, but rather a complex pathogenesis involving all tissues and cells of the joint in which inflammation plays a central role [30]. Therefore, OA patients may benefit little from a single therapy and would likely benefit more from treatments acting on multiple molecular or cellular targets [31]. To this end, combining different drugs could lead to more efficient strategies to combat such a complex, multifactorial disease. The purpose of using multicomponent combinations is to achieve the greatest therapeutic benefit while minimizing toxic side effects. However, because multicomponent combinations may interact at multiple levels and the final effect may be synergistic, additive, or even antagonistic, a thorough evaluation of multicomponent safety and efficacy becomes mandatory to ensure the best therapeutic options.

With this in mind, we proposed to investigate the potential anti-arthritic effects of a combination of five plant bioactives, B, BS, HP, E, and C, which have already been shown in vitro and in preclinical studies to be capable of reducing cartilage damage and joint tissue destruction, but for which no systematic evaluation of their anti-arthritic effects in terms of anti-inflammatory activities and maintenance of bone health, both alone and in combination, had previously been undertaken. 

B is a crude extract of pineapple containing a mixture of proteolytic enzymes with potential anti-inflammatory and analgesic properties [32]. In both synovial fibroblasts and human monocytes, it has been shown to act via a molecular mechanism that inhibits pro-inflammatory transcription factors and the expression of pro-inflammatory mediators [33,34]. The effect of B on joint inflammation has been investigated in clinical trials, with initial encouraging results for the treatment of OA [35,36]. However, not all subsequent studies have shown positive results, and it has been suggested that further studies should be conducted to achieve better conditions of use and a better understanding of the mechanisms of action [37]. HP is a medicinal plant native to Africa that is already approved in Germany for the treatment of degenerative musculoskeletal diseases [38]. HP bioactives have shown anti-inflammatory properties in human monocytes [39] and chondrocytes [40]. In addition, some human clinical studies have shown that extracts from HP significantly improve OA symptoms such as pain and limitation of movement [41]. *Boswellia serrata* is a plant that produces a resin traditionally used to alleviate OA [42]. Exposure of cartilage explants and human articular chondrocytes to BS has been shown to inhibit collagen matrix degradation via a mechanism involving the reduction of MMP-9 and MMP-13 expression [43], and recent evidence suggests anti-inflammatory activities in human monocytes as well [44]. C is a spice whose medicinal properties have been known for thousands of years [45]. Over the past decades, many scientific lines of evidence have accumulated that sheds light on its anti-inflammatory mechanisms of action [46]. The active ingredient of *Curcuma longa* is curcumin, a yellow phenolic pigment, which has shown multiple beneficial effects in different areas of pathology due to its ability to positively influence a variety of signalling pathways and mediators [47]. In terms of OA, curcumin suppresses inflammation in human articular cartilage cells [48] and monocytes [49]. Finally, E is a mixture of closely related triterpene saponins extracted from the seeds of *Aesculus hippocastanum*. The therapeutically active isoform, known as β-escin, exerts pleiotropic pharmacological effects, including anti-inflammatory, antioxidant, and anti-oedematous activities [50,51]. With respect to OA, it has recently been shown to dose-dependently reduce the production of nitric oxide and prostaglandins by lipopolysaccharide-stimulated monocytes and macrophage-like cells. Furthermore, it accordingly decreases the expression of COX-2, IL-1β, and TNF-α in LPS-challenged synoviocytes [17], making it a promising candidate for the treatment of OA. 

Since multicomponent combination potentially interacts at multiple levels, to fully highlight its beneficial effects or eventual antagonizing activities, it is advisable that in addition to chondrocytes and synovial fibroblasts, bioactives are investigated more extensively and comparatively in also those cells that play a role alongside chondrocytes, including monocytes/macrophages and endothelial cells that support leukocyte adhesion and extravasation and that, giving rise to new proangiogenic sprouts, further support monocyte diapedesis and osteophyte formation. For this reason, we decided to study the combination of B, BS, HP, E, and C in monocytes and endothelial cells challenged with TNF-α, a cytokine critically involved in OA pathogenesis [23]. Because chemical interactions can expose patients to potential toxic risk if combinations of multiple constituents are not accurately and precisely evaluated, we first assessed the toxicity profile of each constituent alone and in combination by measuring total cell protein content and MTT activity. For monocytes, we found that at concentrations comparable to physiologically achievable plasma concentrations, there was no toxicity for E [18], B [19], C [20], HP [21], and BS [22]. However, because endothelial cells exhibited higher sensitivity, the bioactives were tested in combination at half the concentrations of each component. In agreement with literature data, TNF-α was able to induce a deep pro-inflammatory gene reprogramming, inducing the expression of a series of pro-inflammatory and pro-angiogenic gene expression in both monocytes [52] and endothelial cells [53]. Exposure of monocytes to each extract before TNF-α stimulation modulated the expression of the tested genes in different ways, with MMP-9 showing greater sensitivity to bioactive exposure, whereas COX-2 and IL-8 showed lower sensitivity and were modulated only by HP, E, and C, and B, HP, and BS, respectively. Similarly, exposure of endothelial cells to each component before TNF-α differentially affected the expression of the genes tested: again, MMP-9 showed higher sensitivity, in addition to COX-2, KDR, and BMP-2, whereas ICAM-1 showed lower sensitivity and was modulated only by E and C. As shown synoptically in Table 2, lower modulatory activity was found for B, which, in agreement with the lowest TPC content and RSA activity, was able to modulate the expression of only 7 genes out of 13 tested. Better efficacy was otherwise observed for E and C, which, according to higher polyphenol content and RSA, were able to modulate the expression of 12 out of 13 genes tested. However, consistent with the hypothesis of a complementary activity exerted by a multi-component combination, even at lower concentrations than stand-alone components, we observed that the Flonat Fast^®^ components together had the ability to significantly curb the expression of all stimulated genes; this suggests that the bioactive combination has the ability to affect OA pathogenesis more integrally.

COXs metabolize arachidonic acid to prostaglandin H2 (PGH2), which is then metabolized by prostaglandin E (PGE) synthase to PGE2, a major mediator of inflammation [54]. COX isoforms include COX-1, which is encoded by prostaglandin endoperoxide synthase (PTGS)-1 and constitutively expressed in many tissues, and COX-2, which is encoded by PTGS-2 and induced by various cytokines and growth factors, including TNF-α [55,56]. By inhibiting the production of prostaglandins, COX-2 inhibitors are now the pharmacological treatment of choice for the management of pain and inflammation in OA [57]. Cyclooxygenase (COX)-2-specific inhibitors such as celecoxib have been used with success in the treatment of OA and have shown few side effects [56]. Targeting COX-2 has therefore become one of the most important goals of pharmacological research for the treatment of OA [57]. For this reason, we evaluated in both monocytes and endothelial cells the ability of Flonat Fast^®^ components to reduce the gene and protein expression of COX-2. Our data confirm the ability of E [17], C [58], and HP [59] to inhibit COX-2 induction in monocytes, but in contrast to what has already been observed for BS [60] and B [34], we did not observe significant modulatory activity. This inconsistency could be due to different cell models and/or different challenging stimuli that may have triggered different intracellular signalling pathways [61]. Similar to monocytes, we also confirm the ability of E [62] and C [63] to modulate COX-2 induction in endothelial cells. However, to our knowledge, our data show for the first time an attenuating effect of B, HP, and BS on endothelial COX-2 expression.

In addition to pain management, COX-2 inhibition is now thought to have broader disease-modifying potential, including preservation of cartilage, synovium, and bone [57]. It was observed that COX-2 inhibition by celecoxib reduced MMP-13 expression in rat OA cartilage and in this way prevented cartilage damage. Under the same experimental conditions that were able to reduce COX-2 induction, we also observed a significant reduction in the expression of MMP-9 in both monocytes and endothelial cells. There is ample evidence that COX-2 is a targetable component of the signalling pathway responsible for the increased expression of proteinases by macrophages [64,65] and endothelial cells [66]. It is possible that the inhibition of MMP-9 expression by Flonat Fast^®^ components is mediated, at least in part, by the inhibition of COX-2. Inhibition of MMP-9 expression by Flonat Fast^®^ components, besides contributing to the prevention of cartilage degradation, may also be in support of a multilevel and multicellular explanation of the anti-angiogenic effects described below [67,68].

Synovitis in OA is pathogenetically characterized by invasion and activation of macrophages and lymphocytes, release of large amounts of pro-inflammatory and catabolic mediators into the joint cavity, and a local increase in vascularization of the synovial membrane [69]. The latter process plays a key role in exacerbating the osteoarthritic process by facilitating the infiltration of immune cells into the synovium [69]. Therefore, synovial membrane angiogenesis and monocyte extravasation represent two important targets for the treatment of osteoarthritis [70]. We clearly observed that exposure of endothelial cells to Flonat Fast^®^ components reduced the expression of adhesion molecules involved in the attraction (MCP-1) and following firm adhesion of monocytes to endothelial cells (VCAM-1, and to a lesser extent ICAM-1) with a functional return expressed in a significant reduction of the number of monocytes adhering to the endothelium. Modulation of endothelial adhesion molecule expression has already been studied with success in the presence of E [71], BS [72], and C [73], but, to our knowledge, no literature reports for B and HP. Our data fully support this evidence, as we did not observe any modulatory effect of B and HP on their own. However, a comprehensive holistic protective potential was evident when the components were administered together. In support of a multilevel and multicellular efficacy that can be achieved by combining all Flonat Fast^®^ components, we also observed, for the first time, a down-regulation of VLA-4, the leukocyte ligand for VCAM-1 and accordingly of the adhesion potential of monocytes.

The interaction of VLA-4 with VCAM-1 supports trans-endothelial chemotaxis of monocytes by facilitating trans-endothelial migration [74]. The reduction in both VCAM-1 and VLA-4 expression argues for inhibition of immune cell entry into the synovium, which, together with inhibition of COX-2 and MMP-9 expression and associated angiogenic sprouts, may contribute to the overall maintenance of synovial health.

However, COX-2-derived prostaglandins are also involved in modulating other cytokine- and growth factor-dependent genes, including those belonging to the TGF-β superfamily [75]. Recent findings have demonstrated for TGB-β signaling pathways a pathogenic role in OA development and progression [76]. In concert to BMP-2 signaling activation [77], its mechanistic role was demonstrated in the regulation of chondrocyte homeostasis during cartilage destruction, in synovia hyperplasia, and, most of all, in the regulation of subchondral bone cell behavior during osteophyte formation, another feature of OA [76]. Accordingly, in a mouse model of OA, injection of the TGF-β1 isoform or BMP-2 has been shown to lead to osteophyte formation in the knee joint [78], while blocking studies with specific TGF-β1 inhibitors have shown that they are able to suppress the same process [79], confirming the role of TGF-β1 in osteophyte formation. These observations clearly demonstrate that the TGF-β/BMP-2 axis plays a dominant role in regulating osteophyte formation, and our results further support the Flonat Fast^®^ components as potential multilevel modulators of OA disease. 

From a pathogenic perspective, the multifactoriality and polygenicity of OA recognize, in the exacerbation of oxidative stress and in the overproduction of ROS, an essential common denominator [80]. The increased production of ROS can damage joint structural biomolecules, and ROS, as an intracellular signaling component, is associated with the induction of numerous pro-inflammatory genes and inflammation-related events in OS, including the infiltration of inflammatory cells, angiogenesis, and osteophyte formation [81,82]. As a result, many efforts have been made to find therapeutics that intercept/reduce the formation of ROS. Flonat Fast^®^ components have expressed different ROS scavenging capacity (B = HP < BS < C < E) and correspondently, we observed the same trend in the gene-regulatory activities. This hypothesis was confirmed by the DCF-DA assay, which clearly demonstrated the ability of Flonat Fast^®^ to affect endocellular ROS levels in both monocytes and endothelial cells. However, which redox-sensitive transcription factors and/or signaling pathways are targeted by Flonat Fast^®^ remains to be elucidated and represents one of the goals of future investigations. 

## 4. Materials and Methods

### 4.1. Chemicals

Flonat Fast^®^ components: B (INCI name: bromelain; botanical name: *Ananas comosus*, was obtained from the pineapple stem and was titrated at 2500 Gelatin Dissolving Units - GDU-/g); HP (INCI and botanical name: Harpagophytum procumbens, was obtained from the root and contained ≥ 10% harpagoside); BS (INCI name: *Boswellia serrata*; botanical name: *Boswellia serrata* Roxb., was obtained from the rubbery resin and contained ≥ 65% boswellic acids); C (INCI and botanical name *Curcuma longa*, was obtained from dried rhizome and contained 96.44% curcuminoids and 71% curcumin); and E (INCI name: Escin; botanical name: *Aesculus hippocastanum* L., was obtained from the bark and was titred at ≥ 15% escin), are recognized food supplements and were provided by Essecore S.R.L. (Essecore, Bari, Italy). Powders were solubilised as follow: B, HP, and E were solubilised in water; BS was solubilised in DMSO; and C was solubilised in ethanol. Each vehicle was added to the culture medium at very low concentrations that were pre-tested for cell toxicity (cell number and morphology, MTT) and cell inflammation and showed no effects. 

Tumour necrosis factor (TNF)-α was purchased from Sigma Aldrich (now under Merck, Darmstadt, Germany). Unless otherwise indicated, all other reagents were purchased from Sigma-Aldrich.

### 4.2. Determination of the Total Phenolic Content

The total phenolic content in each extract was determined using a colorimetric assay according to the Folin–Ciocalteau method [83] with some modifications. Briefly, dry extracts were first solubilised in distilled water or DMSO (as previously indicated) and then distributed in different test tubes before mixing with the Folin–Ciocalteu reagent diluted 10-fold with distilled water. After incubation for 5 min in the dark at room temperature, a 7.5% solution of Na_2_CO_3_ was added and allowed to react for 2 h at room temperature. Absorbance was measured at 725 nm using a reader spectrophotometer against a blank sample (solution with no extract added). Concentrations were calculated from a calibration curve of gallic acid (GA) prepared daily under the same experimental conditions. Results are expressed in µg GA equivalent (GAE)/mg of extract sample.

### 4.3. Antioxidant Activity Assays

The scavenging activity of extracts under investigation was detected using the 2,2′-azinobis-(3-ethylbenzothiazoline-6-sulfonate) (ABTS) assay. Briefly, an ABTS working reagent is prepared by mixing equal quantities of freshly prepared 7 mmol/L ABTS solution with 2.45 mmol/L potassium persulphate solution. The ABTS solution is then mixed with methanol to prepare the working solution. Different concentrations of the sample or standard (control sample) (Trolox) is mixed 1:1 (*v*/*v*) with the ABTS working solution, incubated at room temperature for 6 min, and reading the absorbance at 734 nm. The radical scavenging potential (activity) of ABTS in the sample (percent of inhibition) is evaluated from the standard curve. Activities were expressed as Trolox equivalents (TE)/mg of extract sample.

### 4.4. Cell Culture and Treatment

Human monocytoid THP-1 cells were obtained from the American Tissue Culture Collection (Rockville, MD, USA) and maintained in RPMI 1640 medium supplemented with 2 mmol/L glutamine, 100 mg/mL streptomycin, 100 IU/mL penicillin, and 10% fetal bovine serum (FBS) in a 5% CO_2_ humidified atmosphere at 37 °C. Cell viability was routinely determined by trypan blue exclusion. HMEC-1 were provided by Prof. E.W. Ades (Centre for Disease Control, Atlanta, GA, USA). Primary cells were immortalised by transfection with a PBR-322-based plasmid containing the coding region of the large T antigen of simian virus 40 [84]. Cells were cultured in MCDB-131 medium supplemented with 15% FBS, 2 mmol/L glutamine, 100 mg/mL streptomycin, and 100 IU/mL penicillin, 10 ng/mL epidermal growth factor (EGF), and 0.5% hydrocortisone in a 5% CO_2_ humidified atmosphere at 37 °C. Cells were maintained at a density of 4–7 × 10^5^ cells/mL by subculturing them in fresh medium at a ratio of 1:3–1:5 every 4–5 days. 

For cell treatments, monocytes (1 × 10^6^ cells/mL) were cultured in RPMI 1640 containing 10% FBS and HMEC-1 (at confluence) in MCDB-131 containing 15% FBS in the absence or presence of bromelain (100 μg/mL), Devil’s claw (500 μg/mL), Boswellia (20 μg/mL), curcumin (10 μg/mL), and escin (45 μg/mL) for 4 h before stimulation with 10 ng/mL TNF-α or for 0–24 h. 

### 4.5. Cell Viability

Cell viability was determined by the 3-(4,5-dimethylthiazol-2-yl)-2,5-diphenyltrazolium bromide (MTT) assay, a method based on the ability of viable cells to convert MTT, a soluble tetrazolium salt, to an insoluble formazan precipitate that can be quantified spectrophotometrically. In brief, after treatment with the compounds, alone or in combination, and TNF-α exposure, cells were incubated with MTT (final concentration 0.5 mg/mL) for 2 h, and the formazan products were solubilised with isopropanol. Absorbance was then measured at 490 nm with a microplate reader.

### 4.6. RNA Isolation and Real-Time Quantitative Polymerase Chain Reaction

Total RNA was isolated using TRIzol reagent (Thermo Fisher Scientific, Waltham, MA USA) according to the manufacturer’s protocol. RNA was retrotranscribed using the High-Capacity cDNA Reverse Transcription Kit (Thermo Fisher Scientific, Waltham, MA, USA) on a GeneAmp PCR System 9700 (Thermo Fisher Scientific) under the following conditions: 10 min at 25 °C, 120 min at 37 °C, and 5 min at 85 °C.

Real-time PCR (qPCR) analyses were performed using the CFX96 Touch Real-Time PCR Detection System and software (Bio-Rad, Laboratories, Segrate, Italy). All reactions were carried out in a total volume of 25 μL with 50 ng cDNA, 0.3 pmol/L of primer pair, and 12.5 μL 2 × SYBR Green PCR master mix (Bio-Rad) under the following conditions: 2 min at 50 °C, 10 min at 95 °C, and 40 cycles of 15 s at 95 °C and 1 min at 60 °C. Reactions were executed in duplicate on three independent sets of RNA. Negative controls (without RNA addition) were processed under the same conditions as the experimental samples. The quantifications were performed using the comparative critical threshold method (ΔΔCT), and the GAPDH and 18S genes were used as internal control for normalization. **Gene Symbol** The primer sequences used are listed in Table 3.

### 4.7. Cell Lysis and Immunoblotting

After cell treatment with compounds, alone or in combination, and exposure to TNF-α stimulation, THP-1 were washed twice with cold phosphate-buffered saline (PBS), lysed, and separated electrophoretically on a Mini-Protean (Bio-Rad) precast gel. After electrophoresis, fractionated proteins were transferred onto nitrocellulose sheets (Amersham, Freiburg, Germany), and the resulting membranes were saturated with 5% blocking agent in TRIS-buffered saline (TBS, 20 mmol/L TRIS, pH 7.6, 132 mmol/L NaCl)/0.1% Tween 20 for 1 h at room temperature. The obtained blots were incubated overnight at 4 °C with primary antibodies against: COX-2 (Cayman chemicals, Ann Arbor, MI, USA), or Integrin α4 (Santa Cruz Biotechnology, Dallas, TX, USA), and β-actin (Santa Cruz Biotechnology, Dallas, TX, USA), followed by appropriate horseradish peroxidase-conjugated secondary antibodies (Santa Cruz Biotechnology, Dallas, TX, USA). A chemiluminescence kit was used to visualize the protein bands, which were then quantitatively analysed and normalized to β-actin levels using Scion Image Alpha 4.0.3.2 software (National Institutes of Health, Bethesda, MD, USA).

### 4.8. Cell Surface Immunoassay

Surface expression of cell adhesion molecules on HMEC-1 was measured using a surface enzyme immunoassay (EIA) as previously described [85]. Briefly, HMEC-1 (5 × 10^4^/well) were plated in 96-well flat-bottomed microtiter plates precoated with 0.1% gelatin. Once confluence was achieved, cells were pretreated with each compound alone or in combination for 4 h and then stimulated with 10 ng/mL TNF-α for 18 h. After removal of culture supernatants, nonspecific binding sites were blocked with 0.1% FBS for 30 min. Cells were then exposed to mouse monoclonal antibodies against ICAM-1 and VCAM-1 for 2 h at 4 °C before washing with PBS/0.1% FBS and incubation with a biotinylated goat anti-mouse IgG (Immunotech, a subsidiary of Beckman Coulter, Marseille, France) for 1 h. Cells were then washed thoroughly and exposed to streptavidin-alkaline phosphatase. After a final wash, p-nitrophenyl phosphate substrate was added and absorbance was measured using a microplate reader at a wavelength of 405 nm.

### 4.9. Leukocyte-Endothelial Adhesion Assay

Both THP-1 and HMEC-1 were treated alternately or simultaneously with single or mixed compounds at the indicated concentrations for 4 h and then HMEC-1 were stimulated with 10 ng/mL TNF-α for an additional 18 h. 

To examine how many monocytes attached to the cultured endothelium, 10^6^ cells/mL of THP-1 cells were added to the HMEC-1 monolayers. After 30 min, nonadherent cells were removed by careful washing with M199 medium. Images of HMEC-1 and adherent THP-1 cells were visualized and acquired with a phase contrast microscope (10× objective) connected to a digital camera (EVOS XL Core Imaging System, Thermo Fisher Scientific). Finally, adherent monocytes were counted using the ImageJ program (http://imagej.nih.gov/ij/ (accessed on 20 June 2022)). 

### 4.10. In Vitro THP-1 Chemotaxis Assay

For preparation of endothelial-conditioned medium (ECM), confluent HMEC-1 were treated or not with Flonat Fast^®^ components before TNF-α stimulation. Media were collected under sterile conditions, centrifuged to remove cell debris, and frozen at −20 °C until the chemotaxis assay was performed. The migration of THP-1 cells was studied in a Boyden chamber (Corning purchased through Sigma Aldrich, St. Louis, MO, USA), with the upper and lower chambers separated by a polycarbonate membrane with a pore size of 8 µm. THP-1 monocytoid cells were suspended at a concentration of 2.5 × 10^6^ cells/mL in a chemotaxis buffer consisting of RPMI 1640 with 0.1% BSA and added to the upper chamber, whereas HMEC-1-conditioned medium was added to the lower chamber. After 30 min of incubation at 37 °C, the upper chambers were removed, and migrated THP-1 cells were measured by adding a solution of 5 mg/mL MTT to the lower chamber to a final concentration of 0.5 mg/mL. After 2 h, intracellular purple formazan crystals were solubilized and quantified spectrophotometrically. The involvement of chemoattractants as MCP-1 in monocyte attraction was evaluated by the addition of a solution of recombinant MCP-1 to the upper chamber, which greatly reduced monocyte migration by disrupting the chemoattraction gradient.

### 4.11. Cell Migration Assay 

Migration of HMEC-1 was evaluated using the wound healing assay [86]. HMEC-1 cells were seeded in 6-well plates and grown to confluence in complete medium. After reaching confluence, monolayers were vertically scratched with a sterile 200-μL pipette tip, washed twice, and incubated for 4 h in low-serum media in the presence or absence of Flonat Fast^®^ components before stimulation with TNF-α. After 24 h, cell migration into the damaged area was visualized and photographed under a phase contrast microscope (10× objective) connected to a digital camera (EVOS XL Core Imaging System, Thermo Fisher Scientific). The resulting images were analysed using ImageJ software optimized with specific plugins for automatic detection of the scratched area [87]. The cell-empty area was measured and the treatments were compared. The increase in cell-filled area indicates cell migration.

### 4.12. Measurement of Intracellular ROS Production

Measurement of intracellular production of ROS was performed as previously described [85]. In brief, treated endothelial cell monolayers and THP-1 were stimulated for 30 min with TNF-α in 2% serum-containing medium. Cells were then washed before incubation with 50 µmol/L DCFH-DA in HBSS without phenol red for 20 min at 37 °C. After this step, cells were washed again and suspended in HBSS. Fluorescence was immediately measured with a fluorescence plate reader (Fluoroskan II, Labsystem, Helsinki, Finland) at an excitation wavelength of 480 nm and an emission wavelength of 530 nm. Fluorescence microscopy in 6-well plates has been used in some experiments to qualitatively assess the formation of intracellular ROS in response to treatment with mixed compounds and cytokines. In this case, samples were epi-illuminated with a 100-watt Hg lamp and photographed with 490-nm excitation or 520-nm emission filters.

### 4.13. Statistical Analysis

The results were expressed as means ± S.D. Student’s t-test was used to compare the means between the control group and the compound-treated group. Multiple comparisons were made using one-way analysis of variance (ANOVA) followed by Bonferroni’s post-hoc test. A *p* value of < 0.05 was considered statistically significant.

## 5. Conclusions

We have shown for the first time that the components of Flonat Fast^®^, especially in combination, can effectively curb some pathogenetic features that support OA disease progression, including monocyte diapedesis and adhesion to endothelium, pro-inflammatory angiogenesis, and the synthesis and release of pro-inflammatory and pain mediators.

Given the expanding market for dietary supplements, the fact that they are not usually tested for quality and efficacy before being released to the market raises concerns about quality and safety. Our data indicate that these concerns have been preliminarily addressed, as they show that the original Flonat Fast^®^ components have high qualitative content and potential therapeutic use. 

A limitation of our study is that the results presented were obtained only with human cell culture systems. Therefore, they can be considered helpful to gain mechanistic insights, but cannot necessarily be extrapolated to patients, as only results from clinical studies can provide this level of evidence. Under our experimental conditions, we clearly observe possible preventive effects of Flonat Fast^®^. It follows that there is an obvious need to further characterize and understand these properties in order to translate them into clinically safe nutraceuticals with therapeutic potential. Our results highlighting and enhancing the protective, anti-inflammatory mechanisms of E, B, BS, HP, and C, either alone or especially in combination, justify and reinforce the need to further test these molecules in a more clinical setting.

## Figures and Tables

**Table 1:** Total polyphenols content and free radical scavenger activity of Flonat Fast^®^ components.

Extract	Total Polyphenols Content (µg GAE/mg Dry Extract) Mean ± S.D.	Radical Scavenging Activity (µmol TE/mg Dry Extract) Mean ± S.D.
B	6.29 ± 2.20	1.72 ± 0.50
HP	7.42 ± 0.47	0.66 ± 0.05
E	136.65 ± 19.10	15.28 ± 1.49
BS	43.79 ± 6.81	7.72 ± 1.61
C	694.51 ± 132.58	11.59 ± 1.82

Total polyphenol content was determined by the Folin–Ciocalteau assay; radical scavenging activity was determined by the ABTS method. Values shown are means of triplicate determinations (*n* = 3). The colour intensity is proportional to the polyphenols and radical scavenging activity of each compound tested. GAE: µg gallic acid equivalent; TE: Trolox equivalent.

**Table 2:** Synoptic table of the effects of Flonat Fast^®^ bioactives on gene expression, functional activity, TPC, and scavenging activity.

Cell type			Bioactives	B	HP	BS	E	C	FF
			TPC						
			RSA						
			**Gene**						
**Monocyte**			IL-6						
			IL-8						
			MMP-9						
			COX-2						
**Endothelial cell**			VCAM-1						
			ICAM-1						
			MCP-1						
			COX-2						
			VEGF						
			KDR						
			MMP-9						
			TGF-β1						
			BMP-2						
**Monocyte adhesion**									
			No effect on gene induction						
			Effect on gene induction						
			Blue gradients indicate increase in total polyphenol content (TPC) or radical scavenging activity (RSA)						

**Table 3:** Primer sequences used for qPCR analysis.

	Full Name	Forward Primer (5′-3′)	Revers Primer (3′-5′)	Accession Number
MCP-1/CCL-2	Monocyte chemoattractant protein-1/C-C Motif chemokine ligand 2	CCCCAGTCACCTGCTGTTAT	TCCTGAACCCACTTCTGCTT	NM_002982.3
PTGS2/COX-2	Prostaglandin G/H synthase 2/cyclooxygenase-2	TGCTGTGGAGCTGTATCCTG	GAAACCCACTTCTCCACCA	NM_000963.2
IL-6	Interleukin-6	AGGAGACTTGCCTGGTGAAA	CAGGGGTGGTTATTGCATCT	NM_000600.5
IL-8	Interleukin-8	GTGCAGTTTTGCCAAGGAGT	CTCTGCACCCAGTTTTCCTT	NM_001354840.3
BMP-2	Bone Morphogenetic Protein-2	AGACCTGTATCGCAGGCACT	CCTCCGTGGGGATAGAACTT	NM_001200.4
TGF-β1	Transforming Growth Factor β-1	CACGTGGAGCTGTACCAGAA	GAACCCGTTGATGTCCACTT	NM_000660.7
KDR/VEGFR-2	Kinase Insert Domain Receptor/Vascular Endothelial Growth Factor Receptor 2	AGCGATGGCCTCTTCTGTAA	ACACGACTCCATGTTGGTCA	NM_002253.2
VEGF	Vascular Endothelial Growth Factor	GACACACCCACCCACATACA	TCTCCTCCTCTTCCCTGTCA	NM_001171626.1
MMP-9	Matrix metalloproteinase-9	TTGACAGCGACAAGAAGTGG	GCCATTCACGTCGTCCTTAT	NM_004994.2
ICAM-1	Intercellular adhesion molecule-1	AGACATAGCCCCACCATGAG	CAAGGGTTGGGGTCAGTAGA	NM_000201.2
VCAM-1	Vascular Cell Adhesion Molecule 1	CATGGAATTCGAACCCAAAC	CCTGGCTCAAGCATGTCATA	NM_001078.3
18S	18 ribosomal RNA	AAACGGCTACCACATCCAAG	CCTCCAATGGATCCTCGTTA	NR_003286.2
GAPDH	Glyceraldehyde-3-Phosphate Dehydrogenase	ATGGCCTTCCGTGTCCCCAC	ACGCCTGCTTCACCACCTTC	NM_002046.3

## Data Availability

Data is contained within the article and supplementary materials.

## Supplementary Materials

The following supporting information can be downloaded at: https://www.mdpi.com/article/10.3390/ph15101263/s1, Figure S1: Comparison of B, HP, BS, C, and E activity in relation leukocyte adhesion.
