# Supplementary material for: Analysis of the Anti-Inflammatory and Anti-Osteoarthritic Potential of Flonat Fast®, a Combination of Harpagophytum Procumbens DC. ex Meisn., Boswellia Serrata Roxb., Curcuma longa L., Bromelain and Escin (Aesculus hippocastanum), Evaluated in In Vitro Models of Inflammation Relevant to Osteoarthritis
Source: Pharmaceuticals (Basel). 2022 Oct 13;15(10):1263. doi: 10.3390/ph15101263 (PMC9609228; doi:10.3390/ph15101263)
Supplement: Supplementary file 1 [file pharmaceuticals-15-01263-s001.zip › pharmaceuticals-1906479-supplementary.pdf]

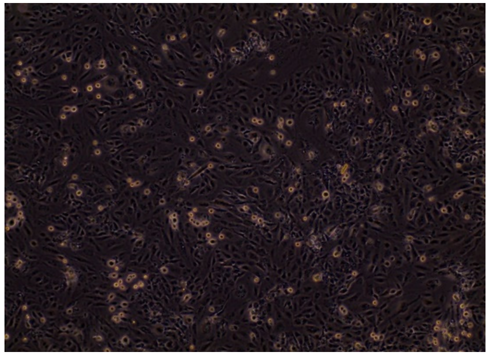

(**A**) Control,
0.61 ± 0.21

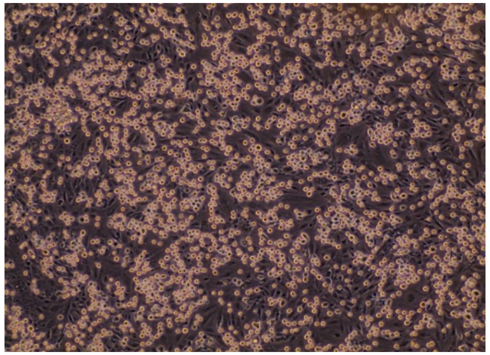

(**B**) TNF-α 10 ng/mL
11.93 ± 0.47#

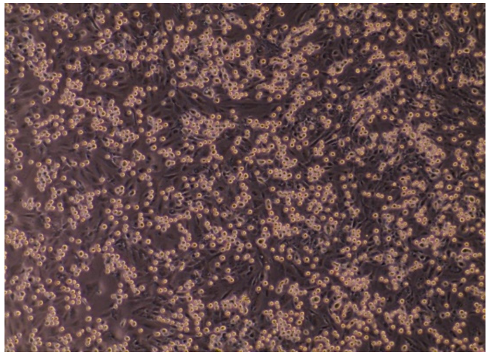

(**C**) B, 50 μg/mL + TNF-α, 10 ng/mL
10.15 ± 1.19

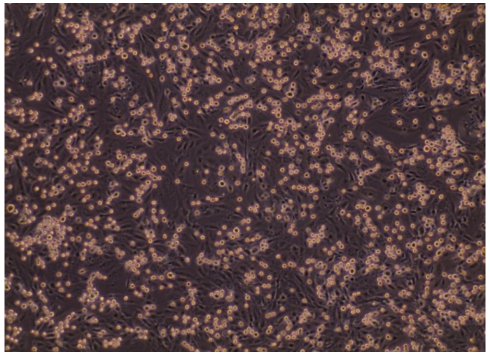

(**D**) HP, 250 μg/mL + TNF-α 10 ng/mL
8.42 ± 0.74*

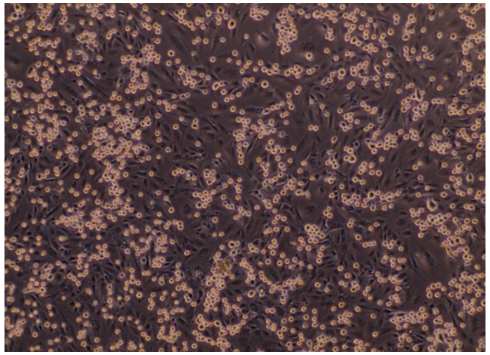

(**E**) E, 22.5 μg/mL + TNF-α 10 ng/mL
7.81 ± 0.63**

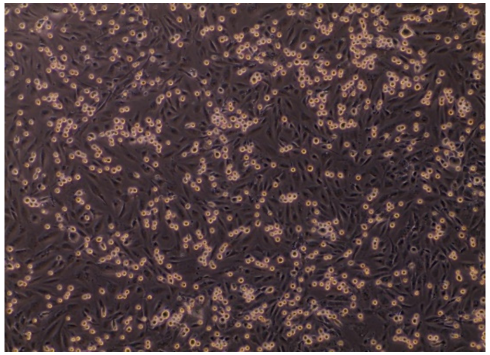

(**F**) BS, 5 μg/mL + TNF-α 10 ng/mL
5.75 ± 0. 84**

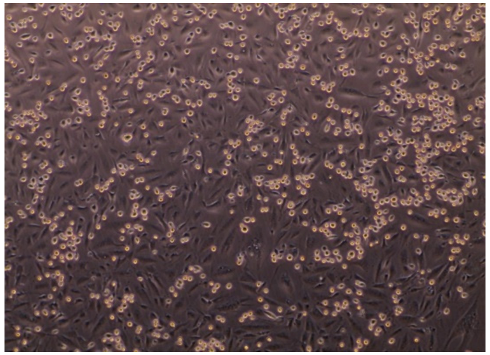

(**G**) C, 5 μg/mL + TNF-α 10 ng/mL
4.66 ± 1.15**

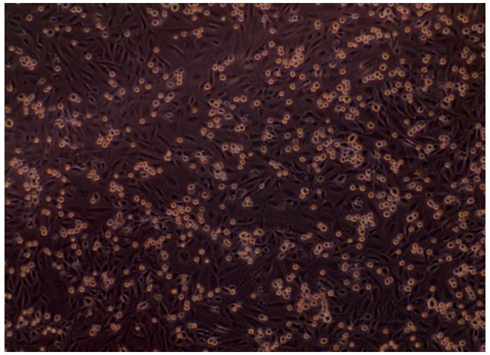

(**H**) FF + TNF-α 10 ng/mL
4.02 ± 0.97**

Figure S1. HMEC-1 were treated with FF components (4 h) alone (from (**C**) to (**G**) or in combination at halved concentration (H) and then either treated with 10 ng/mL TNF-α (from (**B**) to (**H**) or left unstimulated (**A**) for further 18 h. THP-1 (10^6 cells/mL) were then added to the HMEC-1 monolayers. After 1h, non-adhering cells were removed by three washes and images of HMEC-1 and adherent THP-1 cells were visualized and captured with a phase contrast microscope connected to a digital camera. Data (means ± S.D., n = 3) are expressed as number of adherent monocytes per fields. # $p < 0.01$ versus basal (untreated) control; * $p < 0.05$ versus TNF-α alone; ** $p < 0.01$ versus TNF-α alone.
